# Editorial: Boosting positivity by utilizing spirituality as a tool for recovery: post-pandemic process

**DOI:** 10.3389/fpsyg.2023.1251909

**Published:** 2023-08-01

**Authors:** Elif Baykal, Omar Khalid Bhatti, Waqas Farooq

**Affiliations:** ^1^School of Business and Management Science, Istanbul Medipol University, Istanbul, Türkiye; ^2^Hailey College of Commerce, University of the Punjab, Lahore, Pakistan

**Keywords:** spiritual/religious coping, spirituality, pandemic (COVID-19), recovery, positive psychology

As a result of the COVID-19 pandemic, people were confronted with several drastic experiences that led to fear and uncertainty. In traumatic times like the COVID-19 pandemic, spirituality can act as a healing agent for the immune system, helping to promote mental health and pave the way for a more positive psychological state. Many researchers in positive psychology argue that one of the most important roles of spirituality is to promote greater peace of mind and reduce anxiety (Arslan and Yildirim, 2021; Baykal, 2022). Therefore, in this issue, we wanted to show that spirituality can be an important tool for coping with illness and stress during the pandemic.

Undoubtedly, a greater emphasis on spirituality can be helpful in increasing our ability to flourish and heal the traumas suffered during the pandemic. As for positive psychology, the connection with the transcendent and the sense of relief that comes from spirituality can help restore reality and promote resilience (Roberto et al., 2020). In addition, spirituality in the workplace can also be helpful for professionals to adapt to the dynamics of the new normal after the pandemic (Baykal, 2020).

Inspired by the literature on spirituality, we have compiled a number of noteworthy articles in this Research Topic that can be considered good examples of research in the positive psychology of spirituality. The purpose of this issue is to highlight the potential positive effects of spirituality in the aftermath of the COVID-19 pandemic.

There are five articles in this issue, three of which focus on the use of spirituality in the psychological recovery process following the pandemic and two of which, approached with a more positive psychology perspective, focus on the meaning and psychological resources of individuals in coping with stress.

The first work, written by Margaça et al., focuses on the effects of spirituality on coping skills. This work aimed to examine the mediating role of spirituality in the relationship between psychological resilience, optimism, and entrepreneurial success. In addition, the authors sought to uncover possible gender differences. The results of the study show that both optimism and psychological resilience have a positive impact on entrepreneurial success in both genders, but spirituality only in women. This study theoretically and empirically demonstrated that psychological capital and spirituality can be used in entrepreneurial development programs.

Second, Farahani et al. focused on the effects of spiritual struggles on the wellbeing of spiritually sensitive individuals in their work entitled “*The predictors of spiritual dryness among Iranian cancer patients during the COVID*−*19 pandemic*”. They determined the antecedents of spiritual dryness among individuals who had various cancers during the pandemic. They demonstrated the importance of faith and trust in God for patients. They empirically demonstrated the importance of spiritual support for patients during their battle with cancer.

The third article is an opinion piece by Becker. It is titled “*Using the spirituality of funeral rituals for post-pandemic grief recovery*”. This article focuses on the fact that funerals should be considered a psychosocial space where normally socially forbidden emotions can be revealed. This article is insightful in that funerals have the potential to set in motion significant emotional, religious, social, psychological, and meaning-making processes that can rebalance the lives of the bereaved. The funeral of a loved one can evoke deep memories that can impact the mental health of the bereaved. This paper argues that psychologists and psychiatrists should place more importance on traditional family networks and use cultural rituals for the wellbeing of their patients.

In the fourth article, titled “*The relationship between anxiety and depression under the pandemic: the role of meaning in life*”, Shek, Chai, Tan et al. focus on the importance of meaning during the pandemic. The purpose of this article is to examine whether anxiety is related to depression and whether spirituality has the potential to mitigate the association between anxiety and depressive symptoms. Results showed that meaningfulness can mitigate the effects of anxiety on depression, supporting the view that spirituality can serve as a safeguard for psychological morbidity.

Finally, in their article titled “*Stress and depressive symptoms in college students in Hong Kong under the pandemic: moderating effect of positive psychological attributes*”, Shek, Chai, Wong et al. examined the psychological wellbeing of students during the pandemic. They investigated whether positive psychological attributes could moderate the negative effects of stress on depression. The results of the study underscored the importance of positive psychological traits in college students as a protective mechanism for students' tendency toward depression. The results of the study emphasized the function of positive psychological traits in coping with depression.

In this issue, articles with different perspectives have been published that emphasize the importance of spirituality in coping with difficulties in traumatic periods such as COVID-19 and contribute significantly to both the literature on spirituality and the literature on positive psychology. The studies in this issue include both intellectual and empirical studies that make important contributions in demonstrating how spirituality can be used to contribute to human health and wellbeing and therefore may attract the attention of both practitioners and field researchers. The studies in this issue include both intellectual and empirical investigations that make important contributions to how spirituality can be used to contribute to human health and wellbeing, and thus may attract the attention of both practitioners and field researchers. In the papers in this Research Topic, spirituality is approached from a more secular standpoint, remaining within the framework of faith in the Creator without relying on any particular religion. The articles in this issue focus, mainly, on the meaning-making aspect of spirituality in dealing with the pandemic. A focus on the connectedness aspect in future studies may shed more light on the effects of spirituality in coping with difficult situations.

## Author contributions

All authors listed have made a substantial, direct, and intellectual contribution to the work and approved it for publication.
